# Longitudinal Changes in Arthritis Pain Contribute to Subsequent Changes in Body Mass

**DOI:** 10.1093/geroni/igaa057.1393

**Published:** 2020-12-16

**Authors:** Deborah Finkel, Charles Emery, Anna Dahl Aslan

**Affiliations:** 1 Indiana University Southeast, New Albany, Indiana, United States; 2 Ohio State University, Columbus, Ohio, United States; 3 Jönköping University, Jönköping, Sweden

## Abstract

Prior studies have documented that body mass index (BMI) is positively associated with bodily pain. However, data on the temporal sequence of BMI and pain suggest mixed results, with some studies indicating a bi-directional relationship, and other research among older adults supporting a uni-directional relationship from BMI to increased pain. Thus, it is critical to further examine temporal dynamics between changes in BMI and changes in bodily pain to help explicate possible mechanisms influencing the relationship. This study evaluated bivariate dynamic models of longitudinal change (McArdle & Hamagami, 2003) in the relationship between BMI and bodily pain with data from older adults participating in the Swedish Adoption/Twin Study of Aging (SATSA). The sample included 858 individuals aged 45-88 at intake, with up to eight waves of follow-up over 26 years. BMI {weight(kg)/[height(m)]2} was evaluated with objective measures of weight and height recorded by a study nurse. Pain symptoms were measured with six self-report pain questions, reflecting two pain factors: (1) pain in neck, back, or shoulder; and (2) hip pain, history of arthritis, or use of arthritis medications. Results indicated that the relationship between BMI and arthritis pain was uni-directional, with changes in pain symptoms contributing to subsequent changes in BMI, but no evidence that changes in BMI contributed to subsequent changes in pain symptoms. Model comparison indicated that the impact of pain on BMI was greatest before age 70, and then reduced somewhat after age 70, when the impact of other factors on BMI (e.g., ill health) likely increases.

